# Development of a novel patient-reported outcome measure to assess signs and symptoms of COVID-19

**DOI:** 10.1186/s41687-022-00471-w

**Published:** 2022-07-29

**Authors:** Carla Romano, Sheri Fehnel, Jeffrey Stoddard, Jerald Sadoff, Sandy Lewis, Pauline McNulty, Eric K. H. Chan, Emily Evans, Carol Jamieson, Ashley F. Slagle, Allen Mangel, Kelly McQuarrie

**Affiliations:** 1 Patient-Centered Outcomes Assessment, RTI Health Solutions, 3040 East Cornwallis Road, Research Triangle Park, NC 27709 USA; 2 Patient Reported Outcomes, Janssen Global Services LLC, Raritan, NJ USA; 3 Janssen Research & Development, LLC, Raritan, NJ USA; 4 Patient Reported Outcomes, Janssen Global Services LLC, Milpitas, CA USA; 5 Aspen Consulting Services, LLC, Steamboat Springs, CO USA; 6 Patient Reported Outcomes, Janssen Global Services LLC, Horsham Campus, 800 Ridgeview Drive, Horsham, PA 19044 USA

**Keywords:** Patient-reported outcome, COVID-19, Content validity, Signs and symptoms, Patient experience

## Abstract

**Background:**

Given the urgent need for vaccines and treatments for coronavirus disease 2019 (COVID-19), the Symptoms of Infection with Coronavirus-19 (SIC), a comprehensive, patient-reported outcome (PRO) measure of signs and symptoms associated with COVID-19, was developed in full alignment with current US regulatory guidance to support evaluations of vaccines and treatments in development.

**Methods:**

An initial version of the SIC was developed to address concepts identified through a targeted literature review and consultation with experts in infectious diseases and clinicians routinely managing COVID-19 in a hospital setting. A qualitative study was conducted in sites in the United States among 31 participants aged ≥ 18 years who were English-speaking and willing and able to provide informed consent and a self-reported history by telephone or online method. The measure was refined based on additional feedback from the clinicians and three iterative rounds of combined concept elicitation and cognitive debriefing interviews conducted with patients, caregivers, and healthy volunteers.

**Results:**

Among 39 scientific articles identified in the literature review, 35 COVID-19 signs and symptoms were reported and confirmed during interviews with clinicians, patients, and caregivers. Patients and healthy participants suggested changes for refining the draft SIC to ensure consistent interpretation and endorsed both the 24-h recall period and use of an 11-point numeric rating scale (NRS) for capturing change in symptom severity. The final version of the SIC captures the daily presence or absence of 30 symptoms and a rating of severity for 25 of the 30 symptoms using an NRS for those symptoms reported as present.

**Conclusions:**

The SIC comprehensively addresses observations described in the literature, by clinicians, and by patients, and captures patients’ experiences with COVID-19 in a manner that minimizes complexity and facilitates completion for both patients and healthy volunteers. This measure is thus appropriate for use in clinical trials of both therapeutics and vaccines for COVID-19.

**Supplementary Information:**

The online version contains supplementary material available at 10.1186/s41687-022-00471-w.

## Background

In late 2019, a cluster of pneumonia cases in Wuhan, China led to identification of a novel coronavirus, since classified as severe acute respiratory syndrome coronavirus 2 (SARS-CoV-2) [1]. As of March 8, 2022, there have been over 445 million confirmed cases of coronavirus disease 2019 (COVID-19) and nearly 6 million COVID-19-related deaths worldwide [2]. The spread of the disease is concerning, particularly in high-risk populations [3, 4]. The clinical presentation of COVID-19 varies greatly, including asymptomatic infection and mild symptoms, as well as severe manifestations, including pneumonia, respiratory failure, multiple organ dysfunction, and death [5–12]. As understanding of COVID-19 grows, evidence is emerging that it may in fact comprise multiple disease subtypes, based on clusters of symptoms [13].

Though several vaccines have been in use under Emergency Use Authorization, with some obtaining regulatory approval, a greater understanding of COVID-19 signs and symptoms is critical to characterize disease burden and facilitate appropriate care. Patient-reported outcome (PRO) measures can help address these concerns. PROs are particularly important for diagnosis and assignment into treatment pathways, as well as following disease progression, response to treatment, and long-term recovery [14]. Regulators recognize the importance of PRO measures to support the inclusion of patient-focused endpoints for COVID-19 treatment and prevention clinical trials [15].

To our knowledge, few COVID-19-specific PRO measures were described in the literature or were available for use at the time of this study, highlighting a major unmet need [16]. Since this study was initiated, other efforts have been made to develop PRO measures for COVID-19, including the Italian EPICOVID19 Short Diagnostic Scale [17], the COVID-19 Symptom Study in the UK [18], the FLU-PRO Plus [19], and the CDC Coronavirus Self-checker [20]. However, these measures were developed as screening measures or adapted from existing measures of respiratory illnesses; content validity evidence for these measures using rigorous methods as outlined in regulatory guidance documents [15, 21–24] is needed, and the extent to which these instruments are suitable for use in COVID-19 vaccine and treatment trials remain to be investigated. Other non-disease-specific PRO measures have been used, particularly in the assessment of post-COVID syndrome (“long COVID”), but these instruments may not comprehensively capture the full breadth of symptoms, may be burdensome, and may not be meaningful to patients [25–27].

The primary objective of this study was to develop the Symptoms of Infection with Coronavirus-19 (SIC), a PRO measure aiming to assess the presence and severity of COVID-19 signs/symptoms in adults, for use in COVID-19 vaccine and treatment clinical studies. Patient, caregiver, and healthy volunteers participated in concept elicitation and cognitive debriefing interviews to inform item development and refinement of the SIC instrument, to evaluate content validity, and to confirm patient comprehension of the instrument. The inclusion of patients (including those at risk for infection), caregivers, and healthy volunteers was important to capture the patient experience of COVID-19. Instrument refinement was intended to facilitate self-completion.

## Methods

**Fig. 1:**
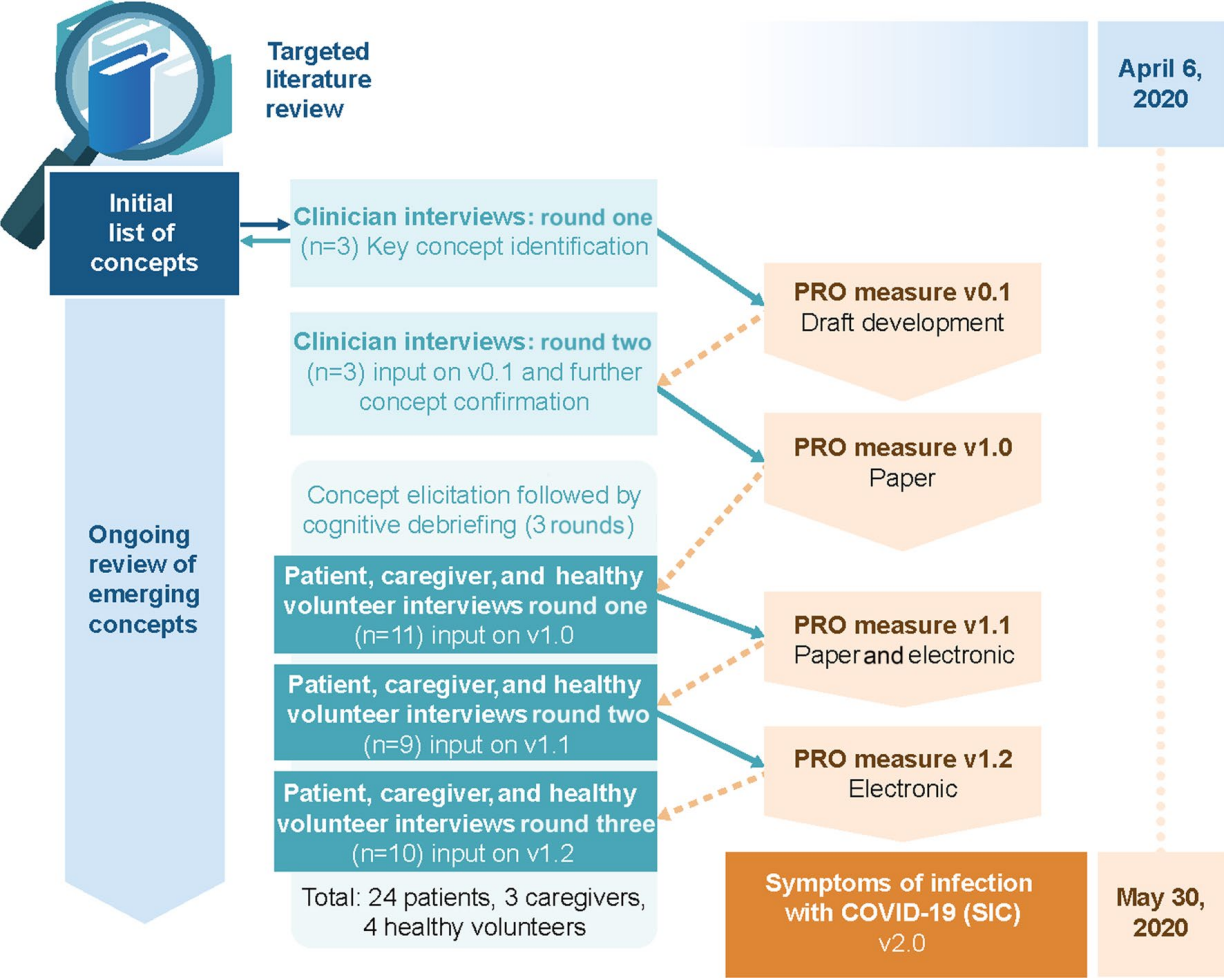
Symptoms of infection with COVID-19 (SIC) development process. Abbreviations: *COVID-19* coronavirus disease 2019, *PRO* patient-reported outcome

To be appropriate for use in vaccine and treatment trial contexts, we sought to create a measure sensitive to both disease onset and changes in key signs and symptoms of COVID-19 using a rigorous process (Fig. 1) in alignment with current regulatory guidance [15, 21–24]. We also sought to balance the need for a fully comprehensive assessment while minimizing length and complexity. The development of this measure was based on the best available evidence as well as anticipating an evolving understanding of COVID-19 signs and symptoms [7, 12, 28].

### Targeted literature review

To identify signs and symptoms of COVID-19, a targeted review of scientific articles published between March 31, 2020, and May 31, 2020, as well as online resources, was conducted. PubMed search terms included “novel coronavirus” or “COVID-19” and “signs” or “symptoms,” and results were limited to human research (Additional file 1: Sect. 1.1). Identified publications and data sources were reviewed in full to identify signs and symptoms of COVID-19 according to body system. The signs and symptoms deemed appropriate for self-report were used to develop the preliminary version of the SIC for discussion with clinicians and evaluation with patients, caregivers, and healthy volunteers.

### Clinician interviews

Three clinicians with experience treating patients with COVID-19 were involved in two rounds of qualitative interviews, including a nurse practitioner assigned to the intensive care unit (ICU) of a large hospital in New York City, a critical care nurse with over 35 years of experience in patient care and manager of the COVID-19 unit in a community hospital in Northeast Pennsylvania, and a pulmonology critical care fellow treating hospitalized patients admitted to the COVID-19 ICU at a university hospital in the midwest. All three had been working in units assigned to triage and treat patients with a diagnosis of COVID-19. Clinician concept elicitation interviews were conducted between April 23–28, 2020. In round one, interviews were conducted to inform development of the draft SIC by identifying signs and symptoms of COVID-19. After the draft SIC had been developed, a second interview gathered feedback related to the content and format of this measure. The SIC review interviews were conducted between May 5–6, 2020, when clinicians were presented with the draft version of the SIC (Additional file 1: Sect. 1.2).

### Item development

**Fig. 2:**
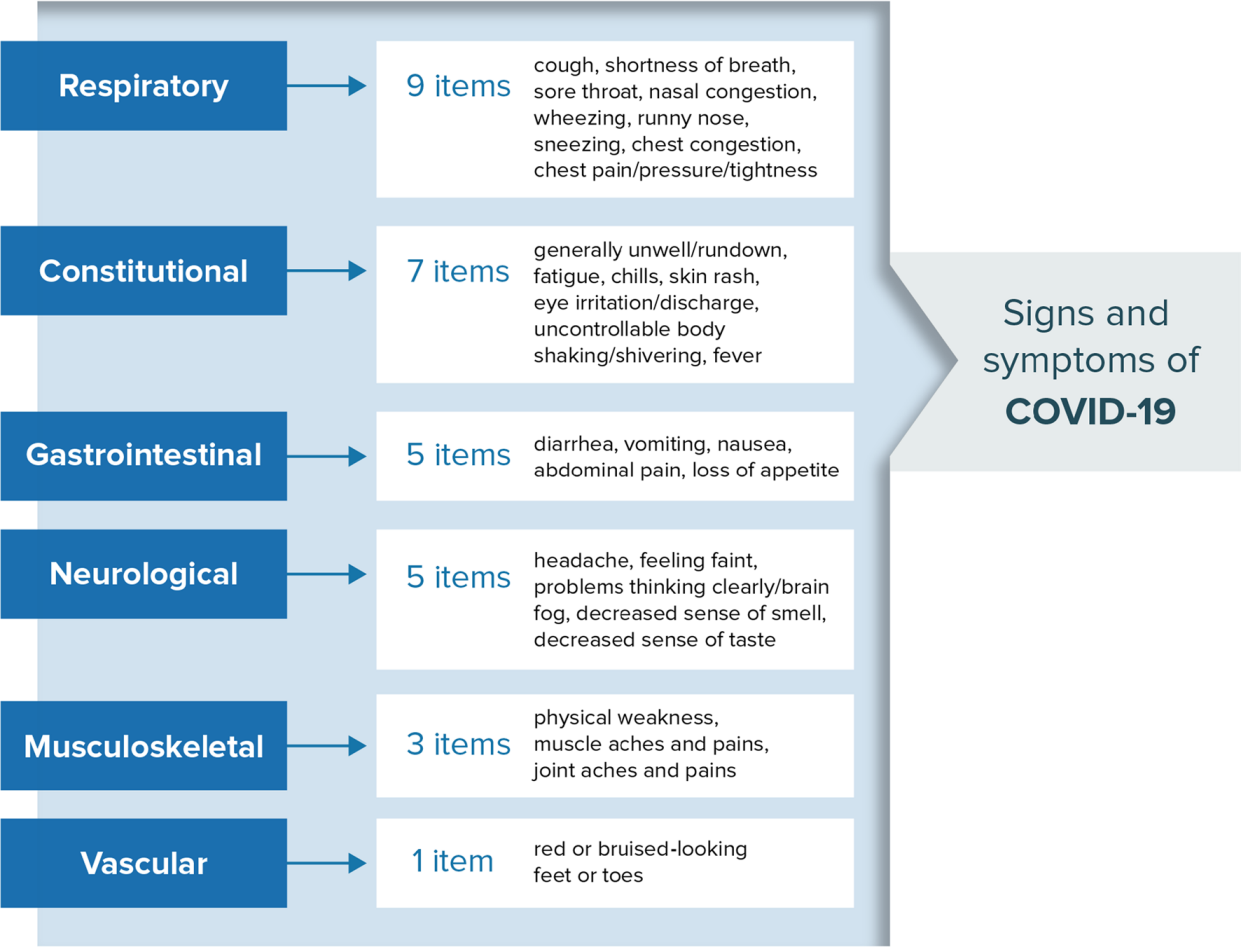
Draft SIC Conceptual Framework. A set of SIC candidate composite scores describing the impact of COVID-19 on different body systems (constitutional, gastrointestinal, musculoskeletal, neurological, sensory, respiratory, upper and lower respiratory) were chosen after performing well in initial assessments. Abbreviations: *COVID-19* coronavirus disease 2019,* SIC* Symptoms of Infection with COVID-19

The initial draft of the SIC (version 1.0) addressed the concepts identified in the literature and confirmed by clinicians (Additional file 1: Table S1). Items were organized based on a conceptual framework (Fig. 2). Items were developed to be succinct and utilize plain language with an appropriate recall period for this context, and with response options that are easily understood and likely to change accordingly with clinical status. A ‘checklist’ approach was utilized where respondents were instructed to determine presence/absence for all signs or symptoms and apply a severity rating only to those items present. This approach was selected to ensure the SIC was comprehensive yet imposed minimal patient burden. In addition to a paper-based version of the SIC, a simple web-based application (i.e., electronic version) was also developed. A link to the web-based version was provided following the fourth interview of round two. The web-based system was designed to be compatible with multiple electronic platforms.

### Patient, caregiver, and healthy volunteer interviews

Three iterative rounds of combined concept elicitation/cognitive debriefing interviews with patients and caregivers were undertaken (Additional file 1: Sect. 1.3 and Sect. 1.4). The first portion of the interview (concept elicitation) was conducted with patients and caregivers to elicit key signs and symptoms of COVID-19, and the second portion (cognitive debriefing) was conducted with patients, caregivers, and healthy volunteers to obtain feedback on the draft versions of the SIC, revised between rounds to ultimately inform development of version 2.0, including the electronic version, which was tested in interview rounds two and three (Additional file 1: Sect. 1.3). Following the incorporation of clinician feedback, patients were recruited to participate in telephone or web-based qualitative interviews to share their experiences with COVID-19 signs and symptoms and to inform refinement of the draft SIC. A qualitative research firm conducted recruitment using prespecified inclusion and exclusion criteria (Table 1). Qualified participating individuals received $100 USD compensation/reimbursement. Caregivers were included to provide additional perspective on the patient experience, including input on how signs and symptoms were observed in the person for whom they provided care. Healthy volunteers were recruited to reflect a vaccine trial population and determine whether the SIC could be accurately completed by people not experiencing symptoms. To identify a representative cohort, additional recruitment targets were applied: inclusion of 15 patients aged ≥ 65 years and 15 patients aged < 65 years; inclusion of ≥ 3 patients previously hospitalized due to COVID-19 within each age group; inclusion of patients both with and without pre-existing comorbidities that put the individual at higher risk of severe COVID-19 (diabetes, hypertension, chronic cardiopulmonary conditions) [3, 4].

**Table 1:** Patient, caregiver, and healthy volunteer inclusion and exclusion criteria

Inclusion criteria	Exclusion criteria
Adult patients (aged ≥ 18 years) self-reporting a positive SARS-CoV-2 PCR test in the 2 weeks prior to screening and experiencing ≥ 2 current, bothersome symptoms due to COVID-19	Participated in a study with any investigational medicinal product in the previous 30 days
**Or**	
Caregivers reporting ≥ 2 COVID-19 symptoms with a positive PCR test for SARS-CoV-2 on behalf of their patient(s)	
**Or**	
Healthy volunteers aged ≥ 18 years	
**AND**	
Willing and able to participate in a 1-h interview via telephone or an online method	
Willing to provide a self-reported medical history	
Able to read, speak, and understand English	
Willing and able to provide informed consent	

Inclusion criteria, including self-reported positive PCR for SARS-CoV-2 and a checklist of current COVID-19 symptoms were presented in the recruitment screener. The most bothersome symptoms were also explored during concept elicitation. Participants described all symptoms they were experiencing and were subsequently asked which were the most bothersome. Consistently, participants distinguished a single symptom that was most bothersome to them, rather than providing a list

Abbreviations: *SARS-CoV-2* severe acute respiratory syndrome coronavirus 2, *PCR* polymerase chain reaction, *COVID-19* coronavirus disease 2019

Patient/caregiver interviews were conducted from May 6–30, 2020. Interviews were one hour in length; audio-recording occurred after informed consent and following participant permission. Before each interview, the lead interviewer conducted an informed consent discussion and obtained verbal participant consent. All interviews were conducted by two members of the project team experienced in conducting qualitative interview research (one lead interviewer who conducted the interview and one scribe/support personnel) using a semi-structured interview guide to ensure consistency of data collection.

### Data analysis

Qualitative analysis of transcripts and field notes was conducted to identify dominant trends in each interview, which were then compared with the results of all interviews to generate themes or patterns in the way participants describe their experiences via constant comparative analysis [29]. The goals of qualitative analyses were to confirm content validity and patient/healthy volunteer comprehension of the SIC, and to document the nature of and rationale for modifications to this measure. A saturation grid of symptoms was constructed to ensure saturation was met.

### Ethics approval

The Institutional Review Board at RTI-International was consulted at the beginning of this study. Using 45 CFR 46 as the ethical framework to guide the review, this study was granted an exemption due to the nature of the participants (independent adults) and minimal risk associated with participation (minimal emotional and no physical risk).

## Results

### Targeted literature review

**Fig. 3:**
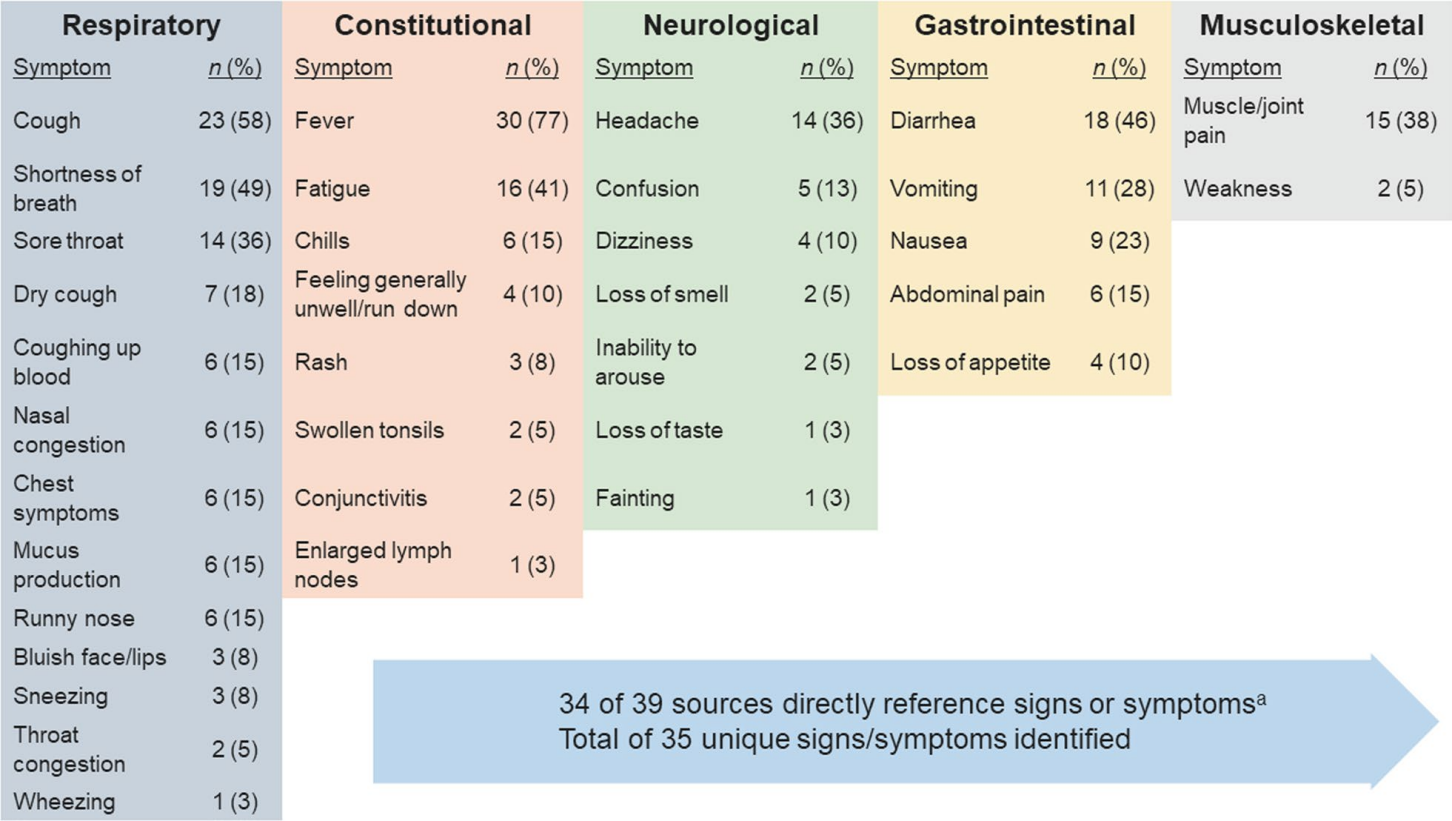
Number of data sources identifying unique signs and symptoms of COVID-19 in targeted literature review. *N* value represents the number of references directly reporting each COVID-19 sign/symptom by body system. ^a^Unique signs/symptoms identified. Abbreviation: *COVID-19* coronavirus disease 2019

In total, 39 articles related to COVID-19 were selected for full-text review (Additional file 1: Sect. 2.1). Among 35 signs or symptoms identified, the most frequently reported were fever (n = 30), cough or dry cough (n = 30), shortness of breath (n = 19), diarrhea (n = 18), fatigue (n = 16), sore throat (n = 14), headache (n = 14), muscle or joint pain (n = 15), and vomiting (n = 11) (Fig. 3). No single, clear constellation of symptoms across patients with COVID-19 was identified. Sign and symptom evaluations differed across the published literature, websites, and websites offering COVID-19 screening and recommendations for testing.

### Clinician interviews (round one)

Clinicians (N = 3) reported symptoms they observed in patients under their care and symptoms reported to them by patients or other healthcare providers caring for the same patients (Additional file 1: Table S1). Uncontrollable body shaking (rigors) and bruised-looking feet or toes were discussed during interviews based on emerging literature at the time of the study. All three clinicians reviewed and confirmed all symptoms based upon experiences providing care or based upon previous consultation with other treating clinicians.

### Item development

Based on the literature and initial clinician interviews, 35 signs and symptoms of COVID-19 were selected for potential inclusion in the SIC. Additionally, based on the emerging literature and clinician reports, three items assessing symptoms of stroke and one to monitor for deep vein thrombosis (DVT) were added before testing with patients. Presence or absence of all signs and symptoms were collected using a checklist “yes” or “no” approach (35 items). For 25 of the 30 items included on the initial version of the SIC, if “yes” was selected, the respondent was asked to rate the severity of the symptom. Based on the literature review and existing symptom-based measures, an 11-point numerical rating scale (NRS; 0 = “none”, 10 = “worst possible”) was selected to capture symptom severity, with a five-point verbal rating scale (none, mild, moderate, severe, very severe) included during the preliminary testing in case anyone expressed difficulty using the 11-point NRS. A 24-h recall period was selected for all items to reduce recall bias and capture symptom variability over time, and to allow for the possibility of important endpoints in clinical studies based on the evaluation of time to symptom resolution or improvement.

### Clinician interviews (round two)

All three clinicians appreciated the use of a patient-reported yes/no checklist approach. The 11-point NRS used to assess symptom severity was endorsed by all three clinicians, noting that this type of scale is typically used for the assessment of pain in a hospital setting and would therefore be familiar and easy for respondents to use. All three clinicians found the draft SIC to be comprehensive and found all items to be suitable for self-report. They also agreed that daily administration with a 24-h recall is appropriate, and that this measure could be useful for screening patients and examining trends of either improvement or worsening of symptoms.

### Patient, caregiver, and healthy volunteer interviews

#### Demographics

A total of 31 individuals participated in an interview: 24 patients, three caregivers, and four healthy volunteers. All caregivers were related to patients who participated in the study and two also contracted COVID-19. Characteristics of patients, caregivers, and healthy volunteers are shown in Table 2.

**Table 2:** Patient, caregiver, and healthy volunteer demographics and characteristics

Patient, caregiver, and healthy volunteer demographics	N = 31
*Type of participant, n (%)*	
Patient	24 (77.4)
Healthy volunteer	4 (12.9)
Caregiver^a^	3 (9.7)
*Gender, n (%)*	
Female	19 (61.2)
Male	12 (38.7)
Age, mean (range)	54.8 (23–76)
*Race, n (%)*	
Caucasian/white	24 (77.4)
African American	4 (12.9)
Mixed race	2 (6.5)
Other	1 (3.2)
*Ethnicity, n (%)*	
Hispanic	3 (9.7)
Not Hispanic	28 (90.3)
*Education, n (%)*	
High school degree or GED	3 (9.7)
Some college	9 (29.0)
Associate degree	2 (6.5)
College degree	7 (22.6)
Postgraduate degree	10 (32.3)
*Employment, n (%)*	
Employed part-time	5 (16.1)
Employed full-time	18 (58.1)
Retired	7 (22.6)
Other	1 (3.2)
Patient characteristics	N = 24
*Patient-reported COVID-19 overall disease severity, n (%)*	
Mild	8 (33.3)
Moderate	14 (58.3)
Severe	2 (8.3)
*Hospitalized due to COVID-19, n (%)*	
Yes	6 (25.0)
No	18 (75.0)
Intubated	1 (4.2)
*Prescribed medications for COVID-19, n (%)*	
Hydroxychloroquine	4 (16.7)
Outpatient antibiotics	3 (12.5)
Oseltamivir	1 (4.2)
*Pre-existing conditions, n (%)*	
Obesity	4 (16.7)
Asthma	4 (16.7)
Hypertension	3 (12.5)
Chronic obstructive pulmonary disease	3 (12.5)
Heart disease	2 (8.3)
Type 1 diabetes	1 (4.2)

^a^Two of the caregivers were related to patients who participated in the study. Two caregivers also tested positive for SARS-CoV-2; although information regarding their symptoms was collected during the interviews, they are not included in the patient counts

Abbreviations: *GED* general educational development, *COVID-19* coronavirus disease 2019, *SARS-CoV-2* severe acute respiratory syndrome coronavirus 2

All patients and caregivers easily recalled and reported symptoms and signs of COVID-19, either as experienced by the participant or observed as a caregiver. General agreement was seen between symptoms reported by caregivers and the patient for whom they provided care (Additional file 1: Sect. 2.2.1).

#### Concept elicitation portion of the interview

##### Signs and symptoms of COVID-19

Patients, healthy volunteers, and caregivers described a wide variety and different sequences of onset of COVID-19 signs and symptoms (Additional file 1: Table S2). Although each patient experienced a mostly unique constellation and progression of symptoms, there were commonalities across patients. Symptoms similar to those of other viral illnesses were reported; however, most patients with COVID-19 described the experience of these common symptoms as different or also described symptoms (eg, loss of taste/smell) that were distinct from any previously experienced viral illness. Patients with underlying conditions easily identified changes/worsening of chronic symptoms (eg, cough, shortness of breath, nasal symptoms) during COVID-19 infection. Fever and cough were the most common initial symptoms of COVID-19 and were among the first three symptoms experienced by half of patients (n = 12 [50%] for each) (Additional file 1: Table S4). Other symptoms commonly experienced at the beginning of infection were fatigue (n = 9, 37.5%), headache (n = 6, 25.0%), muscle aches and pains (n = 6, 25.0%), and feeling unwell (n = 6, 25.0%).

All patients reported experiencing at least one respiratory symptom; most reported ≥ 1 musculoskeletal symptom (n = 23, 95.8%), ≥ 1 neurological symptom (n = 23, 95.8%) and ≥ 1 constitutional symptom (n = 23, 95.8%). Eighteen patients (75.0%) reported ≥ 1 gastrointestinal (GI) symptom and seven patients reported ≥ 1 cardiovascular symptom (Table 3).

**Table 3:** Signs and symptoms reported by patients with COVID-19 (n = 24)

Symptom, n (%)	Spontaneous	Probed	Total
*Respiratory*			
Cough	21 (87.5)	1 (4.2)	22 (91.7)
Chest congestion/mucus production	5 (20.8)	13 (54.1)	18 (75.0)
Shortness of breath	9 (37.5)	7 (29.2)	16 (66.7)
Nasal congestion	4 (16.7)	12 (50.0)	16 (66.7)
Chest pain/pressure/tightness	9 (37.5)	5 (20.8)	14 (58.3)
Runny nose	7 (29.2)	6 (25.0)	13 (54.1)
Sneezing	2 (8.3)	10 (41.7)	12 (50.0)
Sore throat	6 (25.0)	6 (25.0)	12 (50.0)
Wheezing	1 (4.2)	8 (33.3)	9 (37.5)
Mucus in throat	5 (20.1)	3 (12.5)	8 (33.3)
Coughing up blood	0 (0.0)	0 (0.0)	0 (0.0)
*Musculoskeletal*			
Muscle aches and pains	10 (41.7)	10 (41.7)	20 (83.3)
Physical weakness	6 (25.0)	14 (58.3)	20 (83.3)
Joint aches and pains	3 (12.5)	10 (41.7)	13 (54.2)
*Neurological*			
Headache	9 (37.5)	12 (50.0)	21 (87.5)
Loss of smell	9 (37.5)	7 (29.2)	16 (66.7)
Loss of taste	8 (33.3)	8 (33.3)	16 (66.7)
Confusion or “brain fog”	2 (8.3)	12 (50.0)	14 (58.3)
Fainting or feeling faint	1 (4.2)	10 (41.7)	11 (45.8)
Dizziness	1 (4.2)	5 (20.8)	6 (25.0)
Numbness or weakness in arms, hands, or face	2 (8.3)	4 (16.7)	6 (25.0)
Difficulty speaking	0 (0.0)	1 (4.2)	1 (4.2)
Difficulty understanding speech	0 (0.0)	1 (4.2)	1 (4.2)
*Constitutional*			
Fatigue/tiredness	14 (58.3)	9 (37.5)	23 (95.8)
Feeling generally unwell/rundown	6 (25.0)	15 (62.5)	21 (87.5)
Fever	14 (58.3)	5 (20.1)	19 (79.2)
Chills	3 (12.5)	13 (54.2)	16 (66.7)
Uncontrollable body shaking/shivering	1 (4.2)	6 (25.0)	7 (29.2)
Skin rash	3 (12.5)	4 (16.7)	7 (29.2)
Eye irritation/discharge	1 (4.2)	3 (12.5)	4 (16.7)
Bluish lips/face/extremities	0 (0.0)	2 (8.3)	2 (8.3)
*Gastrointestinal*			
Loss of appetite	6 (25.0)	12 (50.0)	18 (75.0)
Diarrhea	5 (20.8)	9 (37.5)	14 (58.3)
Nausea	4 (16.7)	6 (25.0)	10 (41.2)
Abdominal pain	0 (0.0)	8 (33.3)	8 (33.3)
Vomiting	1 (4.2)	4 (16.7)	5 (20.1)
*Cardiovascular*			
Pain, swelling, or redness in legs	0 (0.0)	7 (29.2)	7 (29.2)
Bruised looking feet or toes	0 (0.0)	4 (16.7)	4 (16.7)
*Other*			
Difficulty sleeping	9 (37.5)	NA	9 (37.5)
Visual disturbance	1 (4.2)	NA	1 (4.2)
Liver and kidney pain	1 (4.2)	NA	1 (4.2)

Abbreviations: *COVID-19* coronavirus disease 2019, *NA* not applicable

Fatigue was most commonly reported, experienced by all but one patient (n = 23, 95.8%), followed by cough (n = 22, 91.7%), feeling generally unwell/rundown (n = 21, 87.5%), headache (n = 21, 87.5%), muscle aches and pains (n = 20, 83.3%), and physical weakness (n = 20, 83.3%). In addition to symptoms unique to COVID-19, patients reported that the nature of symptoms common to other respiratory virus infections was a different experience with COVID-19. Multiple body systems could be involved simultaneously as evidenced by the presence of various combinations of respiratory, GI, neurological, cardiovascular, and constitutional symptoms. Overall, except for those with very mild symptoms, most participants described a very high symptom burden. Representative patient descriptions of symptoms experienced are shown in Additional file 1: Table S3.

##### Most bothersome symptoms and disease severity

The most frequently reported bothersome symptoms of COVID-19 were cough (n = 6; 25.0%) due to its persistence and the pain it caused, followed by fever (n = 5; 20.8%), due to the difficulty in controlling this symptom. Loss of taste and smell (n = 3; 12.5%), and fatigue (n = 3; 12.5%) were also reported among the most bothersome. During screening, most patients reported moderate disease severity on a verbal scale. However, as the concept of severity was explored with patients, some verbal ratings of severity were unexpectedly low relative to the severity of symptoms described, as they were comparing themselves with others who had more severe disease or had died. Six patients were hospitalized for COVID-19, including one patient requiring mechanical ventilation, yet only two patients rated their disease as severe.

Symptom persistence appeared to be a key component of COVID-19; multiple patients described the duration of symptoms as much longer than previous viral illnesses, such as influenza. Patients experienced varying courses of disease and varying rates of improvement. Some patients (n = 14) had a limited course of disease, feeling better within a week and returning to normal quickly. Others (n = 9) were sick for at least 4 weeks and still had lingering symptoms at the time of the interview, most notably fatigue and/or physical weakness or upper respiratory tract symptoms. Some patients (n = 20) reported linear symptom progression and improvement, while others (n = 4) reported feeling better one day and then feeling worse again the next day, sometimes repeatedly, with varying symptom presentation. Loss of smell and/or taste were often the last symptoms to emerge as well as the last to resolve.

#### Cognitive debriefing portion of the interview

##### Signs and symptoms of COVID-19

Patients, caregivers, and healthy volunteers provided suggestions for minor wording refinement to ensure consistent interpretation, which were implemented for version 2.0. All participants understood and answered the 25 items requiring a severity rating and the items pertaining to fever, uncontrollable body shaking/shivering, coughing up blood, loss of taste or smell, and red or bruised-looking feet or toes, which did not require a severity rating. Based on clinician input and patient feedback regarding potentially medically emergent symptoms of DVT and stroke, several signs and symptoms were excluded from version 2.0, as patients experiencing these symptoms should be seeking urgent medical care rather than monitoring these symptoms over time in a PRO measure. These included: pain/cramping, swelling, or redness of legs/calves; numbness, tingling, or weakness in your face, arms, or legs; difficulty speaking or forming speech; difficulty understanding speech. In addition, coughing up blood was removed because of a lack of endorsement by patients and clinicians. Finally, the item addressing confusion was removed due to lack of differentiation from the item addressing brain fog and the potential inability to self-report dependent upon symptom severity. One participant suggested adding an item addressing cough-related pain, although this was addressed by the item for chest pain. Other suggestions for additional concepts were judged as beyond the scope of the SIC but may be captured elsewhere, such as mental or emotional impacts of the COVID-19, physical endurance, and comorbidities. Overall, the SIC was refined to 25 items (version 2.0) to be assessed as absent/present, with severity then assessed for symptoms that were present. Five additional items to be assessed only as absent/present were included. Patients endorsed the final measure content, confirming that items in the SIC captured the full spectrum of COVID-19 symptoms they experienced.

##### 11-point NRS and recall period

All participants reported being familiar with an 11-point NRS and indicated that they could easily select a response for each of the severity questions using this scale. Participants did not relate well to the original anchor, “worst imaginable”, and therefore anchors were changed to “none” and “worst possible” to improve clarity. The verbal rating scale was therefore not tested further and the 11-point NRS was selected. A recall period of 24 h was tested with the participants in each round. All participants reported this to be a satisfactory and manageable recall period and considered it appropriate for capturing daily variation in symptoms.

##### Overall impressions

Overall, all participants found the SIC to be relevant to their experience, comprehensive, clear, and easy to complete. Most (n = 20) indicated they would have been able to complete this questionnaire daily during the full course of their infection. All participants reported easily navigating the electronic SIC without difficulty. Additionally, most participants (n = 29) said they would generally prefer electronic administration over paper due to the convenience and accessibility of an electronic version. Representative patient descriptions from the cognitive debriefing are shown in Additional file 1: Table S4.

## Discussion

COVID-19 disease-specific PRO measures, developed according to regulator guidance and best practices, are important for supporting evaluations of vaccines and treatments in development. The SIC was designed to be a comprehensive PRO measure of signs and symptoms associated with COVID-19 in adults and is intended to capture the patient experience of COVID-19. The SIC was developed and refined based upon a targeted review of the literature, expert consultation, and iterative interviews involving concept elicitation and cognitive debriefing with patients, caregivers, and healthy volunteers. The final SIC instrument captures the daily presence or absence of 30 symptoms; of these, 25 symptoms are further addressed for severity using the 11-point NRS if reported as present. The results of the qualitative interviews reported here reflect the highly variable nature of COVID-19, with the majority of participants with COVID-19 experiencing musculoskeletal, neurological, constitutional, and GI symptoms in addition to respiratory symptoms.

Strengths of the study include the iterative nature of interviews and cognitive debriefing and the involvement of experts, patients, caregivers, and healthy volunteers as participants to inform item development, instrument refinement, and confirmation of content validity and patient comprehension of the instrument. Qualitative research is recommended for capturing patient experience necessary to develop patient-centric measures [21].

Limitations of the study include the novel nature of COVID-19 and a corresponding lack of peer-reviewed, large-scale data sets, lack of a fully understood disease trajectory, and lack of ability to identify patients with long-term post-acute symptoms due to the cross-sectional nature of this study. The sample size in this study is in alignment with typical recommendations for qualitative concept elicitation interview studies in clinical outcome assessment development [30]. However, a majority (77%) of participants were White/Caucasian; given that COVID-19 disparately impacts racial, ethnic, and socioeconomically deprived minority groups [31], future studies with more diverse patient populations are needed and have been conducted [32]. Furthermore, COVID-19 diagnosis was based on self-reported data, potentially limiting interpretation of results. While we attempted to interview a population inclusive of patients with comorbidities, the interviews may not be fully representative of patients at high risk for severe COVID-19, and few patients reported severe COVID-19 [3, 4]. Additional qualitative interviews are being conducted to include patient populations who may be at higher risk for severe COVID-19.

The impact of COVID-19 world-wide has been staggering. Development of sensitive and disease-specific PRO measures for COVID-19, such as the SIC, should facilitate regulatory approval of safe and efficacious treatments as well as potentially differentiating various prophylactic vaccines. COVID-19 is increasingly recognized as more than a respiratory disease [7], with some symptoms (respiratory and others) that appear to persist post-infection in approximately one-third of symptomatic patients [33, 34]. The availability of a measure such as the SIC is key for the assessment of long-term outcomes.

Analysis of the data supported that concept saturation was achieved. The final signs and symptoms selected for measurement using the SIC were also consistent with the symptoms of COVID-19 described in the literature at the time of this study [2, 4, 5]. Furthermore, the items selected for the SIC appear to cover the potential for COVID-19 subtypes emerging [13] and lingering post-infection symptoms [33, 34]. As our understanding of COVID-19 signs and symptoms evolved over time, further research has assessed patient experiences with COVID-19. We conducted additional interviews in a diverse and more vulnerable population approximately 1 year after the emergence of SARS-CoV-2, with results consistent with those reported here [32].

Patient-reported ratings of overall disease severity were low in this study, considering the level of symptom severity the patients described. Only two patients (8.3%) rated their experience as severe, yet six patients (25.0%) were hospitalized, with one (4.2%) patient requiring mechanical ventilation. This potentially demonstrates that respondents who recover from COVID-19 may have had their perspective of possible disease severity influenced by the extensive media coverage or experiences with others suffering worse consequences. As a comprehensive PRO measure including use of an NRS to rate individual symptoms, the SIC has the potential to characterize disease severity in a manner that is more accurate and meaningful to patients and clinicians, without limiting respondents to a mild, moderate, or severe designation that may be biased by external factors. In addition, remote monitoring of symptoms using the electronic version of the SIC may help identify patients with severe COVID-19 who are in need of urgent care and those with mild-to-moderate symptoms that can be managed at home and monitored for signs of deterioration; this is particularly important as rapid progression to severe COVID-19 can occur in patients with mild symptoms [16]. The SIC may also enable identification of trends in COVID-19 disease severity and symptom groupings in varying patient demographics. Real-time tracking of self-reported symptoms has been used to predict COVID-19 in the US (eg, COVID-19 Trends and Impact Survey [CTIS]), demonstrating how PROs can be used in public health as an adjunct or substitute for official reporting [35].

## Conclusions

This study has shown that the SIC comprehensively captures the key signs and symptoms of COVID-19 as observed by clinicians and reported by patients with COVID-19, is easy for patients and healthy individuals to complete, and was developed in alignment with current regulatory guidance [15, 21–24]. The SIC also appears to be appropriate for monitoring patients’ symptoms to aid in detection, disease progression, and evaluation of improvement and resolution of symptoms of COVID-19. The comprehensiveness of the SIC and its combination of dichotomous checklist responses and severity ratings across many important symptoms provide flexibility for many uses, including the development of endpoints in clinical studies. The SIC has already been used in large COVID-19 clinical trials [36, 37], and further research has explored applications of the SIC in more diverse populations, including those at-risk for severe COVID-19 [32]. Quantitative research is also underway to evaluate the psychometric properties of the SIC.

## Supplementary Information

**Additional file 1.** Supplementary Methods, Results, and Tables.

## Data Availability

Data are available upon reasonable request.

## Abbreviations

COVID-19: Coronavirus disease 2019
DVT: Deep vein thrombosis
GI: Gastrointestinal
NRS: Numeric rating scale
PRO: Patient-reported outcome
SARS-CoV-2: Severe acute respiratory syndrome coronavirus 2
SIC: Symptoms of infection with COVID-19
