# Supplementary material for: Development of a novel patient-reported outcome measure to assess signs and symptoms of COVID-19
Source: J Patient Rep Outcomes. 2022 Jul 29;6:85. doi: 10.1186/s41687-022-00471-w (PMC9336135; doi:10.1186/s41687-022-00471-w)
Supplement: Supplementary file 1 — Additional file 1. Supplementary Methods, Results, and Tables. [file 41687_2022_471_MOESM1_ESM.docx]

# SUPPLEMENTARY MATERIAL

# SUPPLEMENTARY METHODS

## Targeted literature review

A targeted review of the literature published in PubMed between March 31, 2020, and May 31, 2020, was conducted. PubMed search terms included “novel coronavirus” or “COVID-19” and “signs” or “symptoms,” and results were limited to human research. Early access and pre-print manuscripts, as well as online grey literature, were also used to identify primary data sources. Additional seminal papers or websites were recommended by sponsor staff with relevant expertise to further support this targeted review. Early access and pre-print manuscripts and online grey literature were also used to identify primary data sources. Additional seminal papers or websites were recommended by experts in infectious diseases. The frequency of each sign and symptom was documented and the literature was continuously monitored for emerging signs and symptoms. Given the novelty of coronavirus disease 2019 (COVID-19) and the relevant timescale, it was not possible to perform a conventional systematic review of peer-reviewed studies and meta-analyses. Given the rapid emergence of information supporting new signs and symptoms of infection with COVID-19 in both peer-reviewed and other sources, organization websites, *New England Journal of Medicine*, and *The Lancet* were continually monitored to scan for signs and symptoms not previously identified during the Symptoms of Coronavirus-19 (SIC) development process. Identified publications and data sources were reviewed in full and relevant data were extracted to identify signs and symptoms of COVID-19 according to body system. Among all included sources, the total number reporting particular signs and symptoms was calculated and used to rank the frequency of each sign/symptom. The most commonly reported signs and symptoms were used to develop the SIC for discussion with clinicians and evaluation with patients, caregivers, and healthy volunteers.

## Clinician interviews

Following the targeted literature review, three clinicians routinely treating patients with COVID-19 participated in a qualitative interview. Interviews were designed to inform the development of the draft SIC by identifying signs and symptoms of COVID-19 for assessment. Additionally, clinicians were asked to describe the initial presentation, progression, and resolution of illness, as well as the characteristics of the patients for whom they provide care.

A second qualitative interview was conducted with the same three clinicians to refine the initial draft SIC. This interview focused on gathering feedback related to the content and format of the draft instrument, including the most and least important items from a clinical perspective, as well as input on the questions and the response scale.

## Patient, caregiver, and healthy volunteer interviews

Three iterative rounds of combined concept elicitation/cognitive debriefing interviews with patients and caregivers were undertaken. Round one provided feedback on version 1.0, round two provided feedback on version 1.1, and round three provided feedback on version 1.2. A paper version of the initial draft questionnaire was tested in round one (n=11) and the first half of round two (n=4); an electronic version of the SIC was tested in the remainder of round two (n=5), as well as round three (n=10).

### Concept elicitation portion of the interview

Each of the interviews with patients and caregivers began with a study overview and general questions designed to identify the initial symptoms experienced by patients with COVID-19. Subsequent questions were designed to ensure that all symptoms were identified, to better understand the experience and time course of each symptom, and to explore the relative importance of these concepts to patients.

### Cognitive debriefing portion of the interview

Cognitive debriefing focused on assessing and maximizing the ease of comprehension and completion of the SIC. During cognitive debriefing, participants were asked to describe their thinking as they reviewed each item in the draft SIC. Interviewers also posed follow-up questions designed to further elucidate the participants’ question-answering process, to identify any revisions that might improve patients’ and healthy adults’ understanding of the items (questions and response options), and to determine whether any concepts that are important from the patient perspective were missing from the questionnaire. Respondents were also asked to provide feedback on two global items addressing COVID-19 symptom severity and change, in addition to a surveillance item for use in vaccine clinical studies.

## Sample Patient, Caregiver, and Healthy Volunteer Interview Questions

### Concept elicitation

- When did you first start experiencing symptoms of COVID-19?
  - What did you notice first?
  - How did things change from there?
- What symptoms have been the most bothersome to you? Why?
- Did you seek medical care for your COVID-19 symptoms?
  - What symptoms were you experiencing at that time?
  - Was there a specific symptom for which you decided to seek care?
  - What treatment did you receive? What were the recommendations? What did you try?
  - Did that help? What happened from there?
- <once interview had a complete list of spontaneously reported symptoms>
  - How did your [*symptom*] change over time?
  - How would you describe your [*symptom*] at its worst?
  - How frequently did you experience this symptom?
  - When did your [*symptom*] start to improve?
  - Please describe the course of improvement as well as any setbacks in your recovery from your [*symptom*].

### Cognitive debriefing

- In your own words, what are these instructions asking you to do?
- How could we make these instructions clearer?
- What does the term [insert symptom] mean to you?
- How could we make this item easier to understand?
- If you DO NOT have a particular symptom, how do you respond to the question? [*test skip pattern*]
- How did you choose your answer for this item? (Why was the selected rating chosen?)
- What was your [*symptom*] like when you were feeling the worst?
- What answer would you have chosen when your [*symptom*] first started?

# SUPPLEMENTARY RESULTS

## Targeted literature review

In total, 39 sources of information related to COVID-19 were selected for full-text review (30 in PubMed, nine in the grey literature [newspaper articles, websites, online video presentations, and disease-specific screeners]). A subscription-only clinical resource database, *UpToDate*, was also explored to identify/confirm emergent data. Relevant data were extracted from the full-text papers, websites, and video presentations. Additional papers (n = 7) were reviewed to help characterize the disease and provide background. Additional papers (n = 6) and websites were evaluated throughout the project to confirm the inclusion of the most salient sources.

## Patient, caregiver, and healthy volunteer interviews

### Caregiver interviews

Three caregivers of patients with COVID-19 who participated in an interview agreed to be contacted to complete an interview. Two were spouses and one was a parent of the patients for whom they provided care. Two had tested positive for COVID-19 and shared their own experiences with infection, although the interview focused on the person for whom they provided care. There was general agreement between symptoms reported by caregivers and the person for whom they provided care. All three caregivers reported observing shortness of breath, and one reported specifically being told by the patient’s physician to watch for shortness of breath. Two of the caregivers noted the patients’ reluctance to complain or tell his or her care provider about difficulty breathing. There was no instance of a caregiver being aware of or reporting a symptom not reported by the patient. However, caregivers did describe signs of illness that may have caused more concern to them than the patient, particularly when the patient was described as unlikely to complain.

### Cognitive debriefing

#### Instrument title

The title of the questionnaire presented to participants in rounds one and two was “Symptoms of Infection with COVID-19 (SIC).” All participants understood the title of the questionnaire. No changes were made to the title between rounds one and two. Due to participant feedback and discussions with the sponsor, “COVID-19” was changed to “Coronavirus-19” for testing in round three. All participants in round three understood the title of the questionnaire, “Symptoms of Infection with Coronavirus-19 (SIC).” This title was retained for the final version. No changes were made to the instructions prior to finalization.

#### Additional clinical signs

After cognitive debriefing of the SIC, round one participants were asked about four additional clinical signs related to COVID-19 that might be assessed by patient report: swollen tonsils; enlarged lymph nodes; bluish lips, face, or extremities; and the inability to be aroused from sleep. Most participants thought these signs would be best assessed by a healthcare provider, not the patient. Consequently, associated items were not included in the SIC.

# Supplementary Table 1*.* Signs and Symptoms by Body Class, as Confirmed by Clinicians

| **Symptom, n** | **Observed^a^** | **Confirmed^b^** |
| --- | --- | --- |
| *Respiratory* |  |  |
| Cough | 3 | 0 |
| Shortness of breath | 3 | 0 |
| Nasal congestion | 2 | 1 |
| Chest pain/pressure/tightness | 1 | 2 |
| Runny nose | 1 | 2 |
| Sneezing | 1 | 2 |
| Sore throat | 3 | 0 |
| Throat congestion | 1 | 2 |
| Wheezing | 1 | 2 |
| Coughing up blood | 0 | 3 |
| *Musculoskeletal* |  |  |
| Muscle pain | 3 | 0 |
| Joint pain | 3 | 0 |
| Weakness | 3 | 0 |
| *Neurological* |  |  |
| Headache | 3 | 0 |
| Loss of smell | 1 | 2 |
| Loss of taste | 1 | 2 |
| Confusion | 3 | 0 |
| Fainting | 2 | 1 |
| Dizziness | 2 | 1 |
| Inability to arouse | 1 | 2 |
| *Constitutional* |  |  |
| Fatigue | 3 | 0 |
| Feeling generally unwell/rundown | 3 | 0 |
| Fever | 3 | 0 |
| Chills | 2 | 1 |
| Uncontrollable body shaking/shivers | 1 | 2 |
| Skin rash | 2 | 1 |
| Conjunctivitis | 0 | 3 |
| Bluish lips/face/extremities | 0 | 3 |
| Swollen tonsils | 1 | 2 |
| Enlarged lymph nodes | 1 | 2 |
| *Gastrointestinal* |  |  |
| Loss of appetite | 2 | 1 |
| Diarrhea | 2 | 1 |
| Nausea | 1 | 2 |
| Abdominal pain | 0 | 3 |
| Vomiting | 0 | 3 |
| *Cardiovascular* |  |  |
| Feet or toes look bruised | 1 | 2 |

^a^Observed = sign/symptom observed directly during treatment of patients under care of the reporting clinician.

^b^Confirmed = sign/symptom not directly observed, but clinician was familiar with the sign/symptom from the literature or through discussion with other treating clinicians.

**Supplementary Table 2. Initial Symptomsa Associated with First Disease Onset Reported by Patients With COVID-19 by Body System (N = 24)**

| **Symptom** | **Total (N = 24)** |
| --- | --- |
| Fever | 12 (50.0) |
| Cough | 12 (50.0) |
| Fatigue | 9 (37.5) |
| Headache | 6 (25.0) |
| Muscle aches and pain | 6 (25.0) |
| Feeling unwell | 6 (25.0) |
| Runny nose | 4 (16.7) |
| Chills | 3 (12.5) |
| Sore throat | 3 (12.5) |
| Shortness of breath | 3 (12.5) |
| Nasal congestion | 3 (12.5) |
| Chest pain/pressure/tightness | 3 (12.5) |
| Weakness | 2 (8.3) |
| Trouble sleeping | 1 (4.2) |
| Loss of appetite | 1 (4.2) |
| Loss of smell | 1 (4.2) |
| Nausea | 1 (4.2) |

^a^Patients were asked to report their first three symptoms experienced. Abbreviation: COVID-19, coronavirus disease 2019.

# Supplementary Table 3. Representative Descriptions of Patient Experience of COVID-19 Symptoms

| **Body system** | **Participant description** | **Participant ID** |
| --- | --- | --- |
| Respiratory | *“[Cough] was really bad. It was… I actually had to hold my chest, the middle of my chest, just to cough. Because I was coughing so much, it started hurting the internal parts of my body.”* | 5 |
|  | *“I had this pain in my chest. When I would take a deep breath, it would be this pain, like, my lungs were just hurting.”* | 4 |
| Musculoskeletal | *“This has been over a month already and I still don’t feel like I have the strength back that I had before. It really takes, it really takes your strength away.”* | 11 |
|  | *“I had like muscle aches, joint aches, just lifeless that you can’t even really move around.”* | 6 |
| Neurological | *“Sometimes it would be a simple word and I could not remember it. And it really feels, like the only way I explained it to a friend of mine, it’s like when you would say you’re brain dead. I’m having a weird day, I’m brain dead today. It was like that constantly because you’d forget simple words. So to me, I was like getting nervous because it was affecting my brain. But then when I started reading about it I was like, so other people feel this way so I know this is part of it. So it kind of made me feel better.”* | 3 |
|  | *“I got a different kind of headache. I got a headache that I felt like there was an anvil on my head.”* | 6 |
| Constitutional | *“Well, the fatigue was immense. And I’m just starting now to get the energy back, so the fatigue was very, very, very noticeable. I had no energy. I had absolutely no energy.”* | 9 |
|  | *“There was heat in the house and everything, but I was just catching chills. I was laying down to the point where I had to wrap up with two blankets… Goosebumps were coming all over my body, all over. It was very intense.”* | 2 |
|  | *“I developed, about early to mid-March, a feeling of a low-grade fever associated also with low-grade headaches, a cough, and I went without food or drink for 3 days. I just had no interest in food whatsoever. So those I think were…that's…when I wasn't eating, that seemed to be the first idea that maybe there's something really wrong here and my wife went…I took her to the ER because she also was not feeling well but this was a point in time when there weren't any real testing being done because nobody had enough test kits. So they sent her home and the following morning, the 23rd of March, I got a call from my doctor, my primary care physician and he told me very emphatically that I needed to get to the hospital.”* | 6 |
| Gastrointestinal | *“I just never got hungry. I just said to my wife, we’ll eat, because it’s time to eat, but I’m not really hungry.”* | 14 |
|  | *“I had diarrhoea. I constantly stayed in the bathroom… Or it just ran right through me, and I just had to go to the bathroom.”* | 5 |
| Cardiovascular | *“Now that I’m thinking of that, my feet did like get a little blue, like circulation a little bit, but it’s kind of subsiding now.”* | 3 |
|  | *“Well, my legs would hurt… My calves, like my lower legs, my calves… like a dull ache.”* | 8 |

Abbreviation: COVID-19, coronavirus disease 2019.

# Supplementary Table 4. Representative Descriptions of the Cognitive Debriefing

| **Element** | **Participant description** | **Participant ID** |
| --- | --- | --- |
| Instructions | *“Yes if you have experienced the symptom, and no if you have not experienced in the last 24 hours… Select a rating. Yes, that’s very clear.”* | 7 |
|  | *“It’s very clear to me. To click on yes or no, whether I had a cough, describe how I was with my coronavirus.”* | 8 |
| Scale | *“That’s really the only way you could rate it, one to ten. It couldn’t be mild, severe; one to ten is the better way. Because there’s more of a range.”* | 8 |
|  | *“You could say mild, moderate, severe, but that scale is way too vague. I think numerical is definitely a good way to weigh it.”* | 4 |
| Recall | *“Very easy… The 24 hours should make everything clear, because they’re not asking you for a long period of time. They’re just asking you for the last 24 hours.”* | 5 |
|  | *“If you asked my 24 days, I’m going to have to think it’s different. Twenty-four hours, no, that’s a piece of cake”* | 1 |
| Items | *“I think that covers on a general scale every symptom that you could have or would experience.”* | 6 |
|  | *“Yeah. It’s easy to understand. It’s short, sweet.”* | 8 |
